# Evidence of Ventricular Arrhythmogenicity and Cardiac Sympathetic Hyperinnervation in Early Cirrhotic Cardiomyopathy

**DOI:** 10.3389/fphys.2021.719883

**Published:** 2021-12-08

**Authors:** Shin-Huei Liu, Li-Wei Lo, Yu-Hui Chou, Wei-Lun Lin, Tsung-Ying Tsai, Wen-Han Cheng, Yenn-Jiang Lin, Shih-Lin Chang, Yu-Feng Hu, Fa-Po Chung, Hui-Chun Huang, Shih-Ann Chen

**Affiliations:** ^1^Heart Rhythm Center, Division of Cardiology, Department of Medicine, Taipei Veterans General Hospital, Taipei, Taiwan; ^2^Institute of Clinical Medicine, National Yang Ming Chiao Tung University, Taipei, Taiwan; ^3^Faculty of Medicine, School of Medicine, National Yang Ming Chiao Tung University, Taipei, Taiwan; ^4^Division of General Medicine, Department of Medicine, Taipei Veterans General Hospital, Taipei, Taiwan; ^5^Division of Gastroenterology and Hepatology, Department of Medicine, Taipei Veterans General Hospital, Taipei, Taiwan; ^6^Cardiovascular Center, Taichung Veterans General Hospital, Taichung, Taiwan

**Keywords:** liver cirrhosis, liver sympathetic innervation, cirrhotic cardiomyopathy, cardiac electrophysiology, ventricular tachycardia (VT)

## Abstract

Cirrhotic cardiomyopathy (CMP) is associated with altered cardiac electrophysiological (EP) properties, which leads to the risk of ventricular arrhythmias (VAs). We aimed to evaluate the EP properties, autonomic, and structural remodeling in a rabbit model with early liver cirrhosis (LC). Twelve rabbits were assigned to the sham and LC groups. The early-stage LC was induced by the ligation of the common bile duct. All rabbits received an EP study, VA inducibility test, myocardial, and liver histology staining. Western blot analyses of protein expression and tyrosine hydroxylase stain for sympathetic nerves were performed. The effective refractory period the LC group was significantly longer than the sham group [i.e., left ventricle (LV) 205.56 ± 40.30 vs. 131.36 ± 7.94 ms; right ventricle (RV) 206.78 ± 33.07 vs. 136.79 ± 15.15 ms; left atrium (LA) 140.56 ± 28.75 vs. 67.71 ± 14.29 ms; and right atrium (RA) 133.78 ± 40.58 vs. 65.43 ± 19.49 ms, all *p* < 0.01], respectively. The VA inducibility was elevated in the LC group when compared with the sham group (i.e., 21.53 ± 7.71 vs. 7.76 ± 2.44%, *p* = 0.013). Sympathetic innervation (10^2^/μm^2^/mm^2^) was increased in all cardiac chambers of the LC group compared with the sham group (i.e., LV 9.11 ± 4.86 vs. 0.17 ± 0.15, *p* < 0.01; RV 4.36 ± 4.95 vs. 0.18 ± 0.12, *p* = 0.026; LA 6.79 ± 1.02 vs. 0.44 ± 0.20, *p* = 0.018; and RA 15.18 ± 5.12 vs. 0.10 ± 0.07, *p* = 0.014), respectively. Early LC is presented with an increased ventricular vulnerability, structural heterogeneity, and sympathetic innervation. Close monitoring for fatal arrhythmias is warranted in patients with early stages of LC.

## Introduction

Patients with liver cirrhosis (LC) and cirrhotic cardiomyopathy (CMP) often bear high risks of coronary artery disease and ventricular arrhythmias (VAs). Previous studies revealed that patients with LC may develop dysfunction of the autonomic nervous system (ANS) along with the increased release of norepinephrine vasopressors, which resulted in cardiovascular (CV) complications, such as structural remodeling, higher risks of VAs, and alterations of cardiac electrophysiological (EP) properties (Al Hamoudi and Lee, 2006; Mozos et al., 2011). Clinical studies also showed that the risk of VAs in LC is influenced by the degree of cirrhotic CMP, cholestatic status, remodeling of cardiac ion channel, electrolyte imbalance, increased cytokines, sympathetic hyperactivity, and disturbance in the cardiac electrical and mechanical abnormalities (Moller and Bernardi, 2013; Wiese et al., 2014). Moreover, the appearance of ANS dysfunction is found in the early stages of LC, and the association between sympathetic hyperactivity and VAs may potentially lead to sudden cardiac deaths (SCDs) in patients with LC (Fouad and Yehia, 2014; Kalla et al., 2016).

Cardiac structural and histological remodeling may also result in myocardial heterogeneity that contributes to an unstable cardiac EP property. The relationship and implicated mechanisms between cirrhotic CMP and VAs remain unclear. The aim of the study was to investigate the characteristics and mechanisms of VAs based on the cardiac EP study and structural and ANS remodeling in an animal model with early LC.

## Materials and Methods

### Animal Preparation

Twelve New Zealand male white rabbits (Shulin Breeding Facility, Taiwan), weighing 2.0–3.5 kg, were assigned to the sham group (*n* = 6) and the LC group (*n* = 6). The rabbits were kept at the Animal Center of the Medical Science and Technology Building at the Taipei Veterans Generals Hospital in Taiwan under the guidelines of the Position of the American Heart Association on Research Animal Use (IACUC No. 2019-18). Each animal cage (530 × 630 × 320 mm) housed one rabbit. The rabbits were raised in a soundproof room with consistent temperature (22 ± 2°C), humidity (40–70%), a 12:12 h circadian rhythm, and unlimited access to food (Laboratory Rabbit Diet 5326 HF, PMI, Richmond, IN, United States) and water. All surgical procedures were performed under general anesthesia to minimize suffering.

### Liver Cirrhotic Model and Sham Model

All rabbits were anesthetized by zoletil (i.e., zolazepam and tiletamine) 5 mg/kg plus xylazine 12 mg/kg through intramuscular injection on the thigh. Additional xylazine of 1 mg was given intravenously intermittently to maintain anesthesia when required. Before surgery, all rabbits received blood sample collection and an echocardiogram survey.

All rabbits were placed on the dorsal surface with a 4-cm-long incision over the middle abdominal line into the abdominal cavity. In the sham group, the common bile duct (CBD) was exposed beneath the liver and followed by the closure of the abdominal wound. In the LC group, bile duct ligation (BDL) was performed to induce LC. Supplementary Figure 1 demonstrates the exposed CBD and the location of BDL. Double ligations were performed using 3-0 silk. The first ligation was created near the upper CBD, and the second ligation was created near the lower CBD, followed by dissection between the two ligations (Yang et al., 2015). The BDL was completed by a single operation to minimize the pain from the procedure. Finally, gentamicin sulfate 2.5 g/kg was administrated intravenously after the closure of the abdominal cavity. Pain control was provided by an oral suspension of 1 ml (160 mg/ml) acetaminophen immediately after awakening from anesthesia and 3 ml was mixed with the drinking water of the rabbit for 5 days. All rabbits were matured for 4 weeks before the final experiment. During maturation, complications for cirrhosis including the loss of body weight (BW) and the presence of ascites were monitored. An abdominal sonography was performed weekly to identify ascites.

### Experimental Procedure

After 4 weeks of maturation, all rabbits were arranged for the final experiment. Before the experiment, a warm blank was used for each rabbit to maintain the body temperature. All rabbits were anesthetized by 5 mg/kg zoletil (i.e., zolazepam and tiletamine) plus 12 mg/kg xylazine through intramuscular injection on the thigh. Blood samples were collected for the determination of total bilirubin (T-BIL), alanine aminotransferase (ALT), aspartate aminotransferase (AST), and creatinine (CREA) levels. An echocardiogram was performed on each rabbit. An intravenous line was established for medication and fluid infusion. Endotracheal intubation with connection to a volume cycled ventilator was performed in each rabbit. The exposure of the heart was carried out by thoracotomy (Yamada et al., 2017b). The muscles and ribs were cut carefully, and the bleeding was controlled by radiofrequency coagulation. After the exposure of the mediastinal space, the pericardium was dissected to reveal the entire heart.

### Cardiac Electrophysiological Study and Induction of Ventricular Arrhythmias

All rabbits were arranged for cardiac EP study using a custom-made stimulator (Model 5325, Medtronic, Ltd., Minneapolis, United States) that delivered electrical stimulation pulses of 1 ms in duration to the epicardial surface of the left ventricle (LV), right ventricle (RV), left atrium (LA), and right atrium (RA) through the multielectrode catheter at 2 and 10 times pacing thresholds. Eight S1–S1 300 ms cycles were followed by an extrastimulus. Decremental pacing by 10 ms and then 1 ms until the effective refractory period (ERP) were detected. The shortest S1–S2 interval that resulted in a propagated atrial response was considered as the atrial ERP (AERP). The shortest S1–S2 interval that resulted in a propagated ventricular response was considered as the ventricular ERP (VERP). Finally, ventricular burst S1–S1 pacing (i.e., pacing cycle length decreasing from 250 ms to failure of 1:1 ventricular capture) was performed to induce VA. The VA inducibility was defined by a sustained VA (>30 s) induced by a short ventricular burst S1–S1 pacing for 10 s (i.e., pacing cycle length of 100–150 ms) under maximum pacing output of 10 mA within 10 inductions (Yamada et al., 2017a,b, 2018, 2020).

During the sustained VA, electrical maps of the LV and RV were performed to identify the dominant frequency (DF). The electrical signals were collected using a custom-made 80-electrode contact array (8 × 10 electrodes); each electrode had a size of 300 μm in diameter with an interelectrode distance of 1,500 μm. The VA bipolar and unipolar electrograms of the LV and RV were recorded sequentially on a computer-based digital amplifier/recorder system (Lab System TM PRO EP Recording System, Bard, MA, United States).

### Tissue Collection and Fixation

After the experimental procedure, all rabbits were sacrificed by exsanguination under general anesthesia. The atrial and ventricular myocardial tissues were sampled. The fixation of the myocardial tissue was performed in liquid nitrogen or 20% formalin for tissue preservation. The abdominal cavity was exposed to drain the ascites for measurement and collection of the liver parenchyma. The liver parenchyma was soaked in 20% formalin for fixation.

### Fibrosis and Inflammation Quantification of the Liver Parenchyma

For Western blot analyses, the liver parenchyma was frozen in liquid nitrogen and stored at −80°C. The protein extracts were made by pulverization in a grinder with liquid nitrogen, using a ratio of 1 ml of lysis buffer (phosphate-buffered solution containing 1% Nonidet P-40, 0.5% sodium deoxycholate, 0.1% sodium dodecyl sulfate (SDS), and 0.05% protease inhibitor cocktail solution) (Roche Diagnostics GmbH, Penzberg, Germany) for each 100-mg powdered liver sample. The blots were incubated with antibodies of α-smooth muscle actin (α-SMA, 1:1,000; Abcam plc., Cambridge, United Kingdom), transforming growth factor-β (TGF-β, 1:2,000; GeneTex, Irvine, CA, United States), and tumor necrosis factor-α (TNF-α, 1:1,000; GeneTex, Irvine, CA, United States) for 90 min at room temperature. Next, the blots were incubated for 90 min with the secondary antibody (horseradish peroxidase-conjugated goat anti-mouse immunoglobulin G antibody, diluted with 3% non-fat dry milk in Tris-buffered saline (TBS), 0.1% Tween, Merck KGaA, Darmstadt, Germany), and washed. A subsequent detection of the specific proteins was performed using enhanced chemiluminescence (5-bromo, 4-chloro, 3-indolylphosphate/nitro-blue tetrazolium substrate solution, Amresco Co., Solon, OH, United States). The β-actin (β-actin, Chemicon, Merck-Millipore, Burlington, MA, United States) was used as the internal control. With a computer-assisted video densitometer and digitalized software (Kodak Digital ScienceTM ID Image Analysis Software, Eastman Kodak Co., Rochester, NY, United States), the blots were scanned and photographed; then, the signal intensity (i.e., integral volume) of the appropriate bands were analyzed (Thenappan et al., 2011).

For the determination of hepatic fibrosis, the liver paraffin section was stained with Sirius red staining kit (Polysciences Inc., Warrington, PA, United States). Image J was used to measure the percentage of Sirius red-stained area. For the immunohistochemical staining to detect the liver inflammation, the liver parenchyma was dissected and soaked in 20% formalin. The dissected tissues were stained with hematoxylin and eosin and examined using light microscopy. The immunohistochemical staining study was performed with anti-Cluster of Differentiation 68 (CD68) antibody (diluted 1:200, ab31630, Abcam, Cambridge, United Kingdom) to detect intrahepatic CD68-positive stained macrophages and identify the severity of liver inflammation. The numbers of CD68-positive cells per high-power field (HPF, magnification 200×) were counted using a semi-quantification method (Thenappan et al., 2011).

### Masson’s Trichrome Stain for Fibrosis Quantification

The fibrotic tissues were evaluated using the Masson’s trichrome stain. The myocardium and liver parenchyma were dehydrated by sequential washes with 70, 80, 90, and 100% ethanol. After dehydration, the tissues were fixed in paraffin. For the myocardial tissues, multiple sections were cut parallel from the atrioventricular annulus, including the epicardial and endocardial portion of the myocardium. The Masson’s trichrome stain was achieved with results of blue appearance in the collagen-enriched fibrotic regions. In contrast, the myocardial or hepatic cellular elements would appear in red. Finally, the images of the stained tissues were obtained from multiple slices of tissue stains to calculate the percentage of the collagen region, indicating the degree of tissue fibrosis by an image analyzer [Image-Pro Plus 6.0 (IPP), Media Cybernetics, Inc., Rockville, MD, United States] (Yamada et al., 2017a,b, 2018, 2020). The Laennec staging system was chosen to classify the degree of LC based on histological findings.

### Tyrosine Hydroxylase Stain for Sympathetic Innervation

The sympathetic innervation of the myocardium and the liver parenchyma was identified by immunochemistry staining of tyrosine hydroxylase (TH, PhosphoSolutions Corps., Aurora, CO, United States) (Yamada et al., 2017a,b, 2018). The sympathetic neurons were identified and quantified using the image analyzer (Image-Pro Plus 6.0, Media Cybernetics, Inc., Rockville, MD, United States). The density of the sympathetic nerve was measured as the total area of nerves (μm^2^) per square mm.

### Western Blot Analyses for Protein Expressions of the Myocardium

In the present study, the calcium (Ca^2+^) handling ionic channel proteins, including voltage-gated L-type Ca^2+^ channels (CaV1.2), ryanodine receptor 2 (RyR 2), sarcoplasmic reticulum (SR) Ca^2+^-ATPase 2 (SERCA 2), and Na^+^/Ca^2+^-exchanger type 1 (NCX 1), were investigated (Yamada et al., 2017a,b, 2018, 2020). All myocardial tissues were homogenized and loaded onto a 16% gradient SDS-polyacrylamide gel. The target proteins were transferred through the nitrocellulose filters in the presence of a glycine transfer buffer. Nitrocellulose filters were blocked with 2% albumin in TBS-T buffer for 30 min at room temperature. The nitrocellulose membranes were exposed to the primary antibody in 2% albumin with TBS-T at 4°C. Excess primary antibody was washed away from the nitrocellulose membranes with triple 10-min washes using TBS-T. These membranes were incubated in the enhanced chemiluminescence (ECL) anti-rabbit IgG fragment in TBS-T. After an additional 10-min wash in the TBS-T, the bound antibodies were identified through the Western Blotting Detection System. Finally, expressions of CaV1.2 (Proteintech, IL, United States), SERCA 2 (Thermo Fisher Scientific, Waltham, MA, United States), NCX 1 (Merck KGaA, Darmstadt, Germany), and RyR 2 (Abcam, Cambridge, United Kingdom) were analyzed.

### Statistical Analysis

Continuous data were reported as mean value ± standard error of the mean (SEM). Categorical data were presented as absolute values and percentages. Data variables between the groups (i.e., intergroup) were compared with the Kruskal–Wallis test, and if the *p*-value was <0.05, follow-up comparisons of the different groups were performed with the Mann–Whitney test. Proportion comparisons were performed using the Fisher’s exact test. *p* < 0.05 was deemed statistically significant. The analysis was performed by a senior biostatistician using SPSS statistical software (Version 22.0, SPSS Institute Inc., Chicago, IL, United States).

## Results

### Analyses of Complications, Body Weight, Blood Sample, and Echocardiogram

**FIGURE 1:**
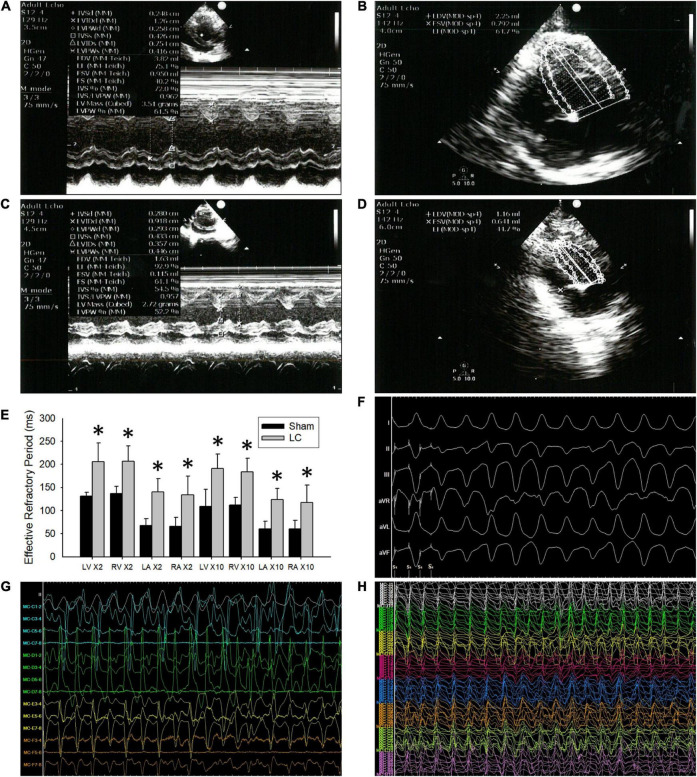
The echocardiograms and cardiac electrophysiological study. **(A,B)** Representative echocardiogram images in the sham group. **(C,D)** Representative echocardiogram images in the liver cirrhosis (LC) group. **(E)** Comparison of 2 and 10 times the pacing threshold for effective refractory period (ERP) (ms) in all the cardiac chambers between the sham and LC group. **(F)** A representative ECG of ventricular arrhythmia (VA) induced by VA inducibility test in the LC group. **(G)** A representative bipolar electrogram of the ventricle during VA in the LC group. **(H)** A representative unipolar electrogram of the ventricle during VA in the LC group. **(E)** Comparison of 2 and 10 times the pacing threshold for effective refractory period (ERP) (ms) in all the cardiac chambers between the sham and LC group. *p < 0.01, compared with the sham group.

In the LC group, all rabbits were presented with complications of cirrhosis including the loss of BW (Table 1) and ascites (mean 29.17 ± 1.94 ml). The BW of the LC group after BDL was significantly lower than the BW before BDL (2.25 ± 0.41 vs. 2.90 ± 0.15 kg, *p* = 0.010). The BW of the LC group was significantly decreased than those in the sham group (Table 1). The serum concentrations of T-BIL, ALT, AST, and CREA in the LC group were significantly elevated compared with those in the sham group (Table 1), respectively. Figures 1A,B are representative examples of echocardiograms in the sham group. Figures 1C,D are representative examples of echocardiograms in the LC group. The LV ejection fraction (LVEF) of the LC group after BDL was significantly lower than the LVEF before BDL (43.20 ± 7.30 vs. 53.97 ± 3.34%, *p* = 0.013). The LVEF in the LC group was significantly decreased when compared to those in the sham group (Table 1).

**TABLE 1:** Comparison of body weight, blood analysis, echocardiogram, and cardiac electrophysiological parameters between two groups.

	Sham (*n* = 6)	LC (*n* = 6)	*P-*value
BW (kg)	2.85 ± 0.17	2.25 ± 0.41	0.029
T-BIL (mg/dl)	0.05 ± 0.04	7.45 ± 1.61	<0.001
ALT (U/L)	25.17 ± 3.31	86.00 ± 50.52	0.032
AST (U/L)	19.17 ± 7.70	123.83 ± 121.61	0.042
CREA (mg/dl)	0.68 ± 0.11	1.32 ± 0.10	0.048
**Echocardiogram**			
LA (mm)	9.72 ± 1.00	9.69 ± 1.07	0.644
IVSd (mm)	2.32 ± 0.21	2.29 ± 0.20	0.648
IVSs (mm)	3.11 ± 0.32	3.10 ± 0.35	0.812
LVIDd (mm)	16.35 ± 0.07	16.29 ± 0.03	0.723
LVIDs (mm)	10.12 ± 0.31	10.09 ± 0.27	0.901
PWd (mm)	2.29 ± 0.19	2.31 ± 0.16	0.651
PWs (mm)	3.51 ± 0.03	3.49 ± 0.06	0.911
Mitral E/A	1.23 ± 0.08	1.25 ± 0.04	0.887
LVEF (%)	54.02 ± 8.50	43.20 ± 7.30	0.011
**Cardiac EP study**			
VA inducibility (%)	7.76 ± 2.44	21.53 ± 7.71	0.013
Bipolar DF (Hz)	8.23 ± 4.41	9.20 ± 7.35	<0.001
Unipolar DF (Hz)	7.27 ± 2.97	7.67 ± 4.47	0.007

*BW, body weight; T-BIL, total bilirubin; ALT, alanine aminotransferase; AST, aspartate transaminase; CREA, creatinine; LA, left atrium; IVSd, diastolic phase of interventricular septum; IVSs, systolic phase of interventricular septum; LVIDd, diastolic phase of left ventricular internal diameter; LVIDs, systolic phase of left ventricular internal diameter; PWd, diastolic phase of left ventricular posterior wall; PWs, systolic phase of left ventricular posterior wall; mitral E/A, mitral valve early to atrial filling velocity ratio; LVEF, left ventricular ejection fraction; EP, electrophysiology; VA, ventricular arrhythmia; DF, domain frequency.*

### Cardiac Electrophysiological Study

In the cardiac EP study, the AERP and VERP at the two times pacing threshold in the LC group were significantly longer than those in the sham group (Figure 1E), respectively. The AERP and VERP at the 10 times pacing threshold in the LC group were significantly longer when compared with those in the sham group (Figure 1E), respectively.

Figure 1F is a representative ECG of VA induced by VA inducibility test in the LC group. The VA inducibility was significantly elevated in the LC group when compared with that in the sham group (Table 1). During DF electrogram mapping, Figures 1G,H are representative bipolar and unipolar electrograms of the ventricle during VA in the LC group, respectively.

### Fibrosis and Inflammation Quantification of the Liver Parenchyma

**FIGURE 2:**
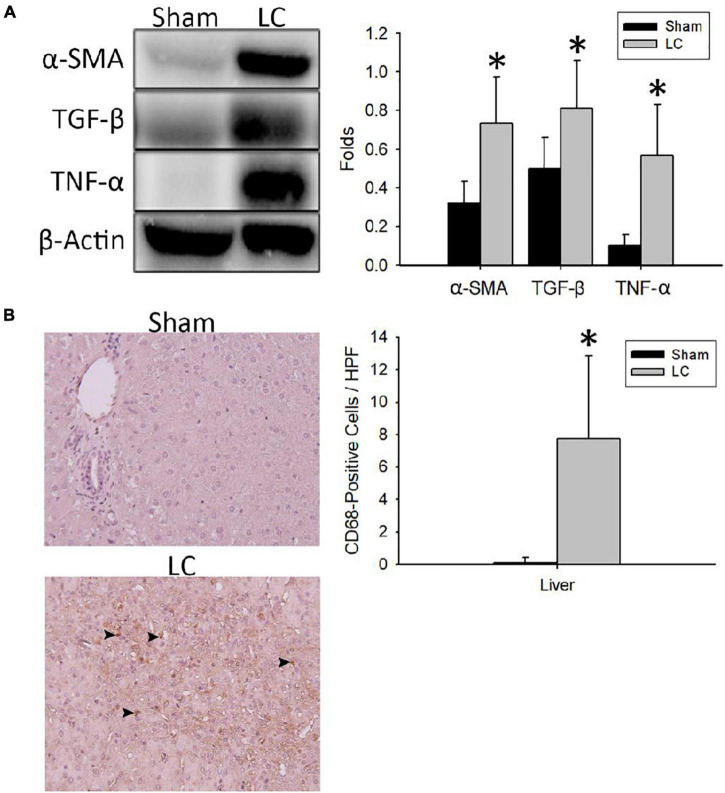
Fibrosis and inflammation quantification of the liver parenchyma. **(A)** Representative Western blot images of protein expressions of fibrosis and inflammation from the liver. The α-SMA and TGF-β levels in the LC group were significantly higher than in the sham group. The TNF-α level in the LC group was significantly higher than in the sham group. **(B)** The left panel shows the representative images of multiple CD68-positive staining cells (denoted in black arrowheads) from the liver parenchyma in the LC group, whereas the sham group revealed negative findings. The right panel shows that the number of CD68-positive cells in the LC group was significantly increased than that in the sham group. *p < 0.01, compared with the sham group. α-SMA, α-smooth muscle actin; β-actin, β-actin; CD68, anti-Cluster of Differentiation 68 antibody; HPF, high-power field; TGF-β, transforming growth factor-β; TNF-α, tumor necrosis factor-α.

Figure 2A shows representative Western blot images of protein expressions of fibrosis and inflammation in the liver parenchyma. For quantification of fibrosis, the α-SMA and TGF-β levels in the LC group were significantly higher than those in the sham group, respectively (Figure 2A). For quantification of inflammation, the TNF-α level in the LC group was significantly higher than that in the sham group (Figure 2A). Figure 2B shows representative images of CD68-positive staining cells from the liver parenchyma in the sham and LC group. The LC group showed a significantly increased CD68-positive cells in the liver parenchyma (Figure 2B).

### Masson’s Trichrome Stain for Fibrosis Quantification

**FIGURE 3:**
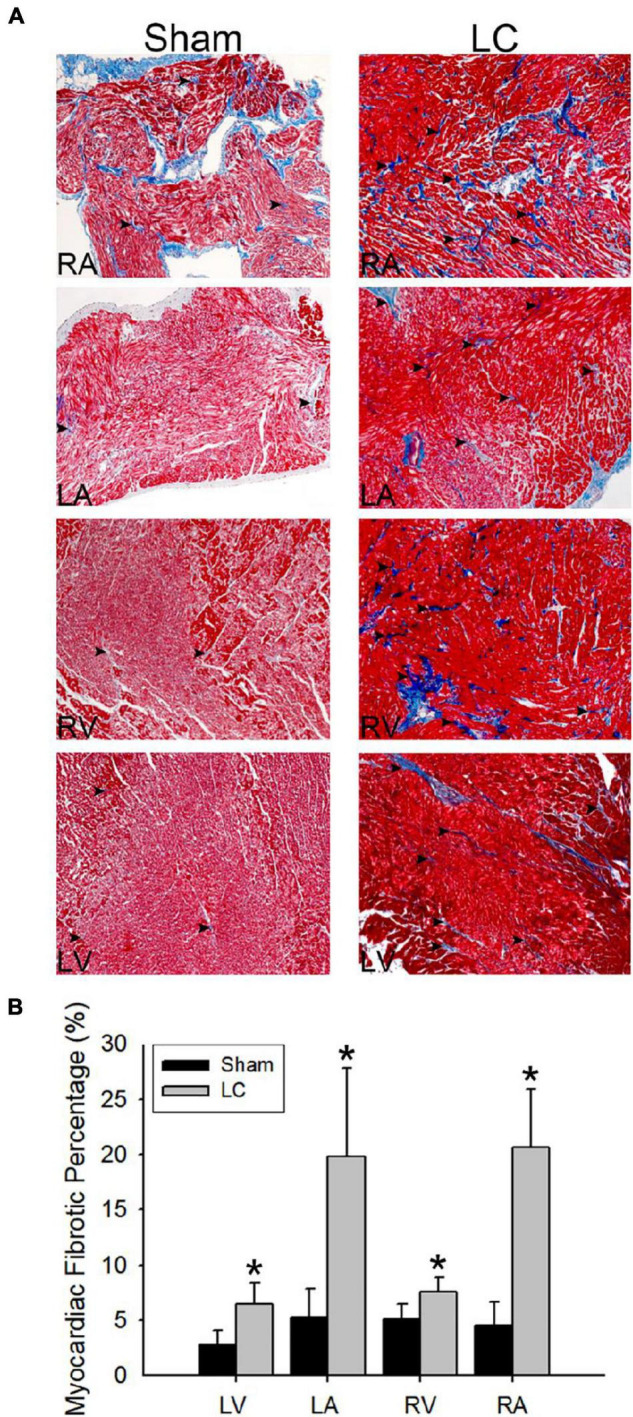
Masson’s trichrome stain for fibrosis quantification of the myocardium. **(A)** The Masson’s trichrome stain (magnification 200×) of the atrial and ventricular myocardium in the sham and LC groups. The red color indicates the normal myocardium. The blue color indicates the fibrotic tissue (denoted in black arrowheads). **(B)** The comparison of percentages of myocardial fibrotic area from Masson’s trichrome stain between the groups. *p < 0.01 compared with the LC group.

Figure 3A illustrates the Masson’s trichrome stain of the collagen deposition in the atrial and ventricular myocardia of the LC group. The atrial and ventricular myocardial fibrosis in the LC group was significantly higher than those in the sham group (Figure 3B), respectively. The LC group revealed significant fibrosis in the liver parenchyma when compared with that of the sham group (3.18 ± 1.68 vs. 1.11 ± 0.21%, *p* = 0.019). The degree of fibrosis and inflammation in the LC group suggested an early stage in the process of LC.

### Tyrosine Hydroxylase Stain for Sympathetic Innervation

**FIGURE 4:**
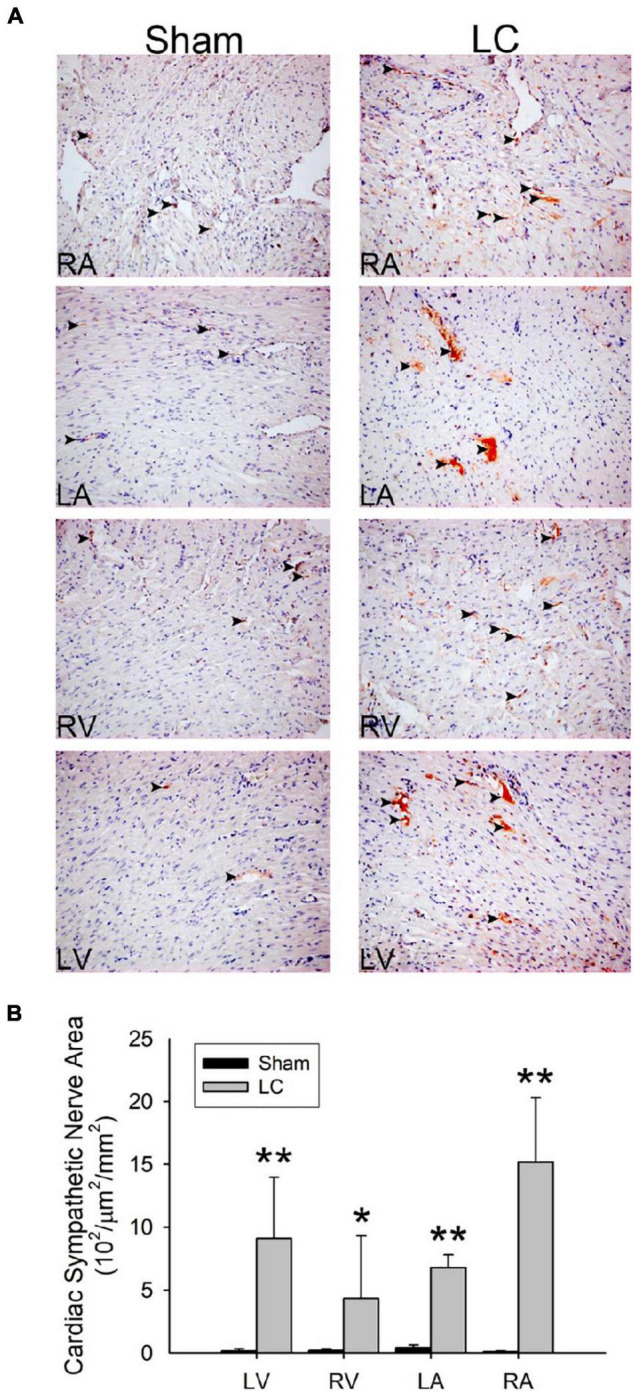
Tyrosine hydroxylase stain for sympathetic innervation of the myocardium. **(A)** The immunohistochemistry stain (magnification 200×) for tyrosine hydroxylase of the atrial and ventricular myocardium in the sham and LC groups. The tyrosine hydroxylase stain showed an increased sympathetic innervation (denoted in black arrowheads) in the LC group compared with the sham group. **(B)** The comparison of the total area of sympathetic innervation in the myocardium between the sham and LC groups. **p < 0.01, compared with the sham group. *p < 0.05, compared with the sham group.

Figure 4A shows the TH stain for sympathetic innervation of the myocardium in both the sham and LC groups. The sympathetic innervation was significantly increased in the bilateral atria and ventricles of the LC group when compared to those in the sham group, respectively (Figure 4B). The LC group showed a significantly increased sympathetic innervation of the liver parenchyma when compared with that of the sham group [0.91 ± 0.83 vs. 0.17 ± 0.13 (10^2^/μm^2^/mm^2^), *p* < 0.01].

### Western Blot Analyses for Protein Expressions of the Myocardium

**FIGURE 5:**
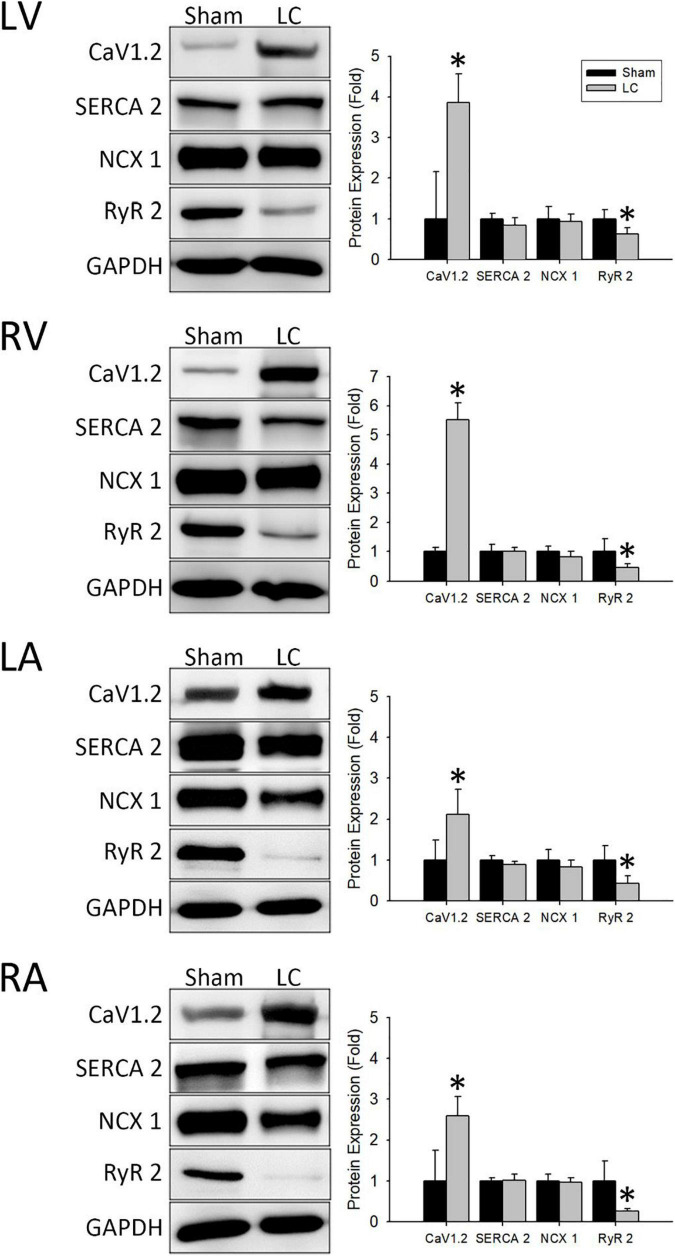
Western blot analyses for protein expressions of the myocardium. (Left) Representative Western blot images of ion channel protein expressions from the left ventricle (LV), right ventricle (RV), left atrium (LA), and right atrium (RA). (Right) The ionic channel protein level of CaV1.2 in the LC group was significantly higher than in the sham group in all cardiac chambers. The ionic channel protein level of SERCA 2 and NCX 1 showed no difference between the two groups in all cardiac chambers. The ionic channel protein level of RyR 2 in the LC group was significantly lower than the sham group in all cardiac chambers. *p < 0.01 compared with the sham group. CaV1.2, voltage-gated L-type Ca^2+^ channel; GAPDH, glyceraldehyde-3-phosphate dehydrogenase; NCX 1, Na^+^/Ca^2+^-exchanger type 1; RyR 2, ryanodine receptor 2; SERCA 2, sarcoplasmic reticulum Ca^2+^-ATPase 2.

Figure 5 (left) demonstrates representative immunochemistry stains with the expression of Ca^2+^ handling proteins in each cardiac chamber of the sham and LC groups. The CaV1.2 levels in each cardiac chamber of the LC group were significantly higher than the sham group (Figure 5, right), respectively. The protein expression of SERCA 2 and NCX 1 in each cardiac chamber revealed no differences between the sham and LC groups (Figure 5, right), respectively. The RyR 2 levels in each cardiac chamber of the LC group were significantly lower than those of the sham group (Figure 5, right), respectively.

## Discussion

### Main Findings

The main findings of this study were as follows: (1) The AERP and VERP of all chambers in the LC group were significantly longer, indicating the prolonged conduction velocities resulted from structural remodeling and myocardial fibrosis; (2) sympathetic innervation was significantly increased in bilateral ventricles and liver parenchyma of the LC group, which could cause sympathetic hyperactivity, resulting in a higher risk of VAs in subjects with LC; (3) increased myocardial fibrosis in the LC group indicated the involvement of structural cardiac remodeling leading to LV systolic dysfunction began in the early stages of LC; and (4) the increased CaV1.2 protein expression and decreased RyR 2 protein expression in the LC group demonstrated intrinsic electrical remodeling and increased cardiac dysfunction in the early stage of LC.

### Cardiac Structural Remodeling in Liver Cirrhosis

Cirrhotic CMP includes myocardial fibrosis, impaired cardiac contractility, altered cardiac relaxation, and EP anomalies. In animal and clinical studies, collagen deposition in the myocardial tissue was found in subjects with cirrhotic CMP, which led to myocardial hypertrophy, systolic, and diastolic dysfunctions (Glenn et al., 2011). The attenuated systolic and diastolic functions can blunt the heart response to stress stimuli by decreased stroke volume and preload reserve, which further contributed to body fluid retention. The imbalanced preload and afterload are more evident in the presence of ascites resulting in a vicious cycle. Our findings were compatible with these studies and have further addressed that the onset of the cardiac structural remodeling can and occur since the early stage of LC.

### Cardiac Electrical Remodeling in Liver Cirrhosis

Several EP anomalies have been addressed in the subjects with LC, including ERP prolongation, cardiac electrical, and mechanical remodeling. In advanced LC, these electrical and mechanical dissociations have been documented in over 60% of subjects with LC and could be reversed to normal conditions after the liver transplantation (Josefsson et al., 2014). A previous animal study suggested the underlying mechanisms included the electromechanical uncoupling or the alterations of cardiac ion channels, but the exact mechanisms and clinical relevance to potential VA or SCD were seldom investigated (Ward et al., 2001). Our study provided additional information on the characteristics of cardiac EP in the LC animal model. In the results from our study, prolonged ERPs indicated prolonged repolarization caused by myocardial structural remodeling, which harbors arrhythmic substrate for conduction disturbance and the potential for scar-mediated tachycardia (Tse et al., 2017). Despite being in the early stages of LC, our study revealed that cirrhotic subjects are vulnerable to fatal VAs due to a lowered threshold for triggered activity in the ventricular myocardium. The finding of these EP anomalies corresponded to several clinical studies that highlighted the concerns of VA risks and SCD in patients with LC (Zambruni et al., 2006; Mozos, 2015).

### Interaction Between Cardiac Calcium Channels and Arrhythmias

In general, the mechanisms of cardiac contraction are driven by action potential duration (APD) in association with the Ca^2+^ influx cascade (Landstrom et al., 2017). The occurrence of arrhythmias in pathological conditions depends on electrical remodeling and altered APD in the cardiomyocytes (Zhang et al., 2016). In the cardiomyocyte, the influx of Ca^2+^ through the voltage-gated Ca^2+^ channels (VGCC) regulated the APD, which triggers cardiac contraction. Among the various types of VGCCs, the CaV1.2 is predominant in the heart. The influx of Ca^2+^ through the CaV1.2 channels triggers a larger release of Ca^2+^ from RyR2 on the SR followed by altered afterdepolarization, resulting in electrical activation and cardiac contraction. During cardiac relaxation, the Ca^2+^ is eliminated from the cytosol by transporting into the SR by the SERCA 2 channel or into the extracellular space by the NCX 1 channel. Under pathological conditions, an altered cascade of Ca^2+^ dynamics could lead to cardiac electrical remodeling, myocardial fibrosis, arrhythmogenic substrate, and cardiomyocyte death (Harvey and Hell, 2013).

Under sympathetic stimulation or in a diseased heart, a phosphorylation cascade leads to an elevated expression of CaV1.2 channels with an increased influx of Ca^2+^ (Harvey and Hell, 2013). In our study, the LC group showed a significant elevation in the expression of CaV1.2 protein with a compensatory response of decreased expression of RyR 2 protein. This finding indicated that the intrinsic electrical remodeling of Ca^2+^ channels began at an early phase of LC. This finding also corresponded to the increased sympathetic innervation of the myocardium, which stimulated the increased expression of CaV1.2 channels in the LC group. Furthermore, the increased activation of CaV1.2 channels contributed to Ca^2+^ overload, which could potentially lead to a higher risk of arrhythmia and myocardiocyte death (Zhang et al., 2016). In contrast, the SERCA 2 and NCX 1 channel proteins showed no difference between the sham and LC groups, demonstrating that the myocardial relaxation was not altered during the early stage of LC.

### Sympathetic Hyperactivity in Liver Cirrhosis

The previous studies revealed that ANS dysfunction is commonly found in advanced LC with clinical features of autonomic neuropathy and is a poor prognostic indicator for patients with LC (Frith and Newton, 2011). In cirrhotic subjects, the activated renin-angiotensin-aldosterone system led to the increased levels of circulating catecholamine, downregulation of β-adrenaline receptor signaling in multiple organs, and various hyperdynamic states, resulting in sympathetic hyperactivity (Wiese et al., 2014). Clinical evidence has demonstrated that sympathetic hyperactivity causes myocardial injury and altered intracellular ion fluxes, such as L-type Ca^2+^ and potassium (Na^+^) channels, leading to cardiac dysfunction (Ruiz-del-Arbol and Serradilla, 2015). Basic studies have proven that an altered ANS may influence the change in voltages of the cellular membrane and intracellular ion-exchange, leading to unstable electrical activities and arrhythmias (Huang et al., 2017). Moreover, the increased catecholamine levels caused remodeling of ion channels, altered after depolarization, and altered APD in cardiomyocytes, which could potentially initiate VAs (Huang et al., 2017). Basic and clinical studies have proven the association between sprouting of the sympathetic nerve and VAs, which further supported the potential risk of SCD. The myocardial sympathetic innervation heightens the sympathetic tone resulting in increased ion current through ion channels, which creates a more excitable substrate for initiating VAs (Chen et al., 2001).

Our study demonstrated an increased sympathetic innervation in the myocardia of all four cardiac chambers and the liver parenchyma in the LC group. The microscopic findings of sympathetic neurons suggested that sympathetic hyperactivity began in the early stage of LC, which gradually developed into ANS dysfunction with an increased risk of VAs. Our findings supported the interaction between sympathetic hyperactivity and ventricular arrhythmogenicity.

### Clinical Importance

The cardiac ANS in subjects with cirrhotic CMP contributes to the pathophysiological changes in the myocardial contraction and conduction abnormalities (Wiese et al., 2014). The clinical assessments of autonomic neuropathy are limited to non-invasive examinations, such as heart rate variability, baroreceptor reflex, and head-up tilting test, but could only provide indirect information (Freeman, 2006). Clinically, patients with cirrhotic CMP lack direct quantitative measurements that could facilitate the management of cardiac ANS dysfunction. Moreover, the investigations on cardiac EP abnormalities in cirrhotic CMP are limited, and cirrhotic patients with jaundice are less likely to receive invasive cardiac EP studies under jaundice. Therefore, this basic research provided an additional evidence on the underlying mechanisms of cardiac ANS and EP dysfunction in the cirrhotic CMP. Despite our current findings, further investigations are required to address the current treatment obstacles.

## Conclusion

Rabbits with early LC had increased vulnerability to VAs through structural heterogeneity, regulation of myocardial ion channels, and sympathetic remodeling. Since the latent risks from an arrhythmogenic ventricular substrate are often neglected in patients with LC, close monitoring for potentially fatal arrhythmias is warranted even in the early stages of LC.

## Data Availability Statement

The original contributions presented in the study are included in the article/Supplementary Material, further inquiries can be directed to the corresponding author/s.

## Ethics Statement

The animal study was reviewed and approved by the Taipei Veterans General Hospital.

## Author Contributions

S-HL, L-WL, and H-CH designed and initiated the project. S-HL, H-CH, Y-HC, and W-LL were responsible for the laboratory experiments, animal care, and data analysis. S-HL was responsible for manuscript preparation. All authors provided critical comments during the experiment and approved the final manuscript.

## Conflict of Interest

The authors declare that the research was conducted in the absence of any commercial or financial relationships that could be construed as a potential conflict of interest.

## Publisher’s Note

All claims expressed in this article are solely those of the authors and do not necessarily represent those of their affiliated organizations, or those of the publisher, the editors and the reviewers. Any product that may be evaluated in this article, or claim that may be made by its manufacturer, is not guaranteed or endorsed by the publisher.

## Supplementary Material

Supplementary Figure 1 Bile duct ligation in the liver cirrhosis model. The ligation of bile duct was performed to induce liver cirrhosis in rabbits. The star represents the liver and the asterisk represents the duodenum that was connected to the common bile duct (CBD). The black arrows represent double ligations using 3-0 silk at the two ends of the CBD. The dotted line represents the dissection line between the two ligation sites.
