# Supplementary figures and images for: Evidence of Ventricular Arrhythmogenicity and Cardiac Sympathetic Hyperinnervation in Early Cirrhotic Cardiomyopathy
Source: Front Physiol. 2021 Dec 8;12:719883. doi: 10.3389/fphys.2021.719883 (PMC8692789; doi:10.3389/fphys.2021.719883)

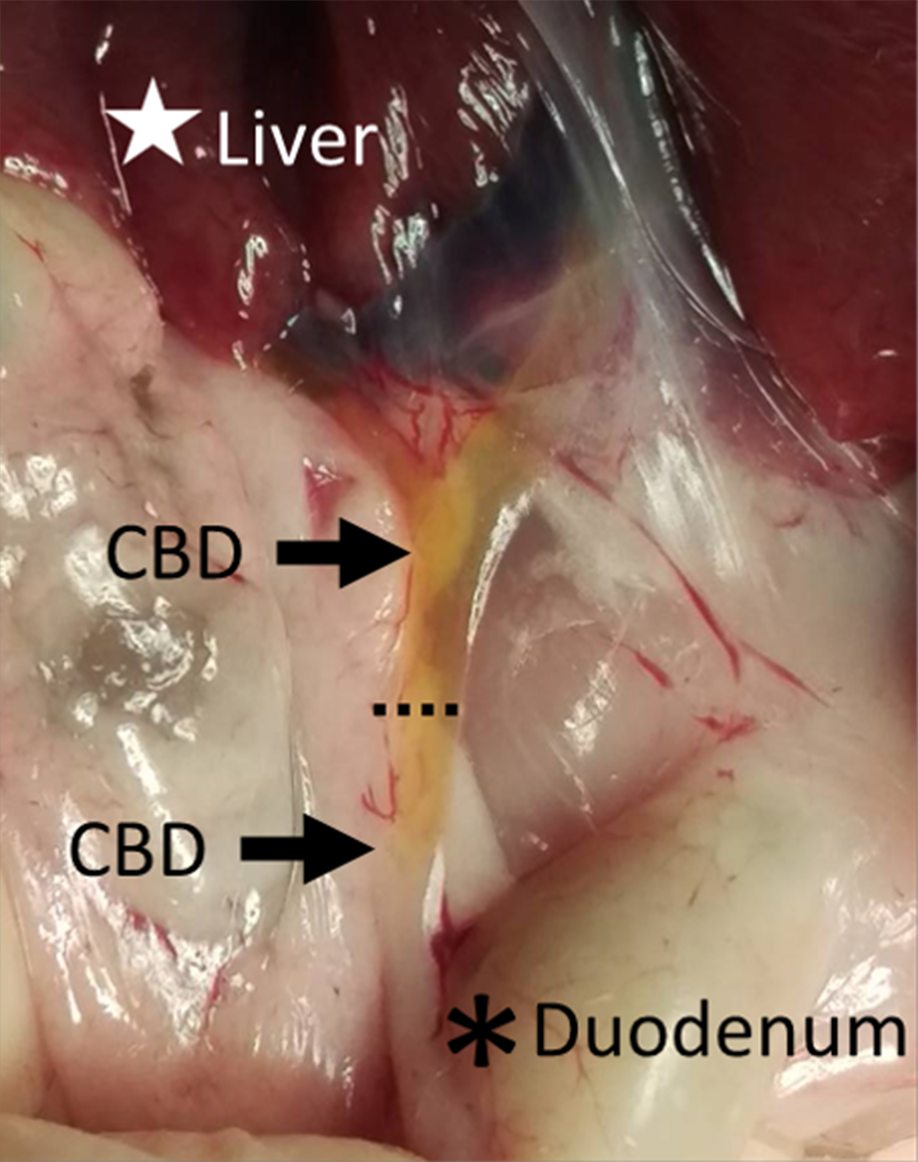

Supplement: Supplementary Figure 1 — Bile duct ligation in the liver cirrhosis model. The ligation of bile duct was performed to induce liver cirrhosis in rabbits. The star represents the liver and the asterisk represents the duodenum that was connected to the common bile duct (CBD). The black arrows represent double ligations using 3-0 silk at the two ends of the CBD. The dotted line represents the dissection line between the two ligation sites. [file Image_1.TIF]
